# Resident-Run Clinic for Nonsurgical Injectable Facial Aesthetics: A 5-Year Institutional Experience

**DOI:** 10.1093/asjof/ojag068

**Published:** 2026-04-21

**Authors:** Sydney Somers, Kaylee B Scott, Ashraf Patel, Chase Hart, Alexandra Vitale, McKenna Waller, Rachelle Clarke, Aaron Dadzie, Devin Eddington, Cori Agarwal, Courtney Crombie

## Abstract

**Background:**

Resident-run clinics (RRCs) have become an important component of plastic surgery training. This study evaluates outcomes from a longitudinal RRC focused on neuromodulator and soft tissue filler injections over a 5-year period.

**Objectives:**

The aim of this study was to assess clinical outcomes, patient retention, resident experience by postgraduate year (PGY) level, and the safety of RRCs as a training model.

**Methods:**

A retrospective review of patients seen in our institution's RRC from January 2020 to September 2024 was performed. Variables included demographics, injectable type and anatomic location, revision rate (overall and by PGY), complication rate, patient retention, and surgical conversion.

**Results:**

A total of 380 patients (92.1% female, mean age 41) accounted for 1168 encounters (mean 3.3 visits/patient). Thirty-seven residents participated during the study period. Neuromodulator, filler, and combined treatments comprised 55.3%, 20.9%, and 23.7% of encounters, respectively. Corrugators (91%) and lips (74%) were the most common neuromodulator and filler sites. The overall complication rate was 0.17% (infection and eyebrow ptosis), and the revision rate was 5.2%, most commonly because of perceived ineffectiveness (58.7%) or asymmetry (38.1%). Revision rates decreased with advancing PGY (PGY-1: 9.0% vs PGY-6: 3.0%). Sixty-five percent of patients returned for at least 1 subsequent visit, and 5.5% later scheduled surgery with a resident or attending.

**Conclusions:**

Nonsurgical RRCs provide a safe, effective training environment for progressive autonomy in aesthetic injectables while maintaining low complication and revision rates comparable to other practice settings.

**Level of Evidence: 4 (Risk):**

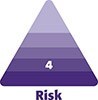

Resident-run clinics (RRCs) have become integral to aesthetic plastic surgery training, offering residents opportunities to develop hands-on experience performing cosmetic procedures.^1,2^ Approximately 60% to 70% of plastic surgery residency programs have dedicated RRCs, which emphasize resident autonomy while addressing the growing need for comprehensive, competency-based residency training models.^3-5^ Studies have demonstrated that RRCs are valuable for both patients and residents.^6-8^ For patients, RRCs offer cosmetic procedures at a discounted rate, reducing the cost burden associated with these treatments. Residents benefit from a semi-autonomous learning environment that fosters the development of technical and nontechnical skills, enhances professionalism, and increases practice accountability. However, there is considerable variability in the integration of aesthetic surgery training across programs, and competency in nonsurgical aesthetic procedures, including cosmetic injectables, remains a significant challenge.^2,7,9-12^ Additionally, there are limited studies specifically evaluating the clinical outcomes and long-term patient retention within these nonsurgical RRC.^1,13^

Neuromodulator and filler injections are the most popular minimally invasive cosmetic procedures, comprising 68% of all reported aesthetic interventions in the United States.^4,14,15^ Competition for administering cosmetic injectables has increased drastically with nonplastic surgeons representing a significant share of the market space for these procedures. In a survey study, the American Society of Plastic Surgeons (ASPS) and The Aesthetic Society found that patients are just as likely to seek minimally invasive procedures from nonplastic surgeons.^16^ Consequently, it is important for plastic surgery trainees to receive comprehensive training and exposure to cosmetic injectables to meet rising patient demand, remain competitive in practice, and deliver safe, high-quality outcomes.

At our institution, a weekly RRC integrates residents of all training levels, providing experience with neuromodulators and soft tissue fillers while emphasizing graduated autonomy. This study analyzes 5 years of data from our institution's RRC, including patient demographics, injectable volume, revision rates, and patient retention. By assessing the educational and clinical value of RRCs, our study aligns with the American Council of Academic Plastic Surgeons’ recommendations for standardized training in nonsurgical aesthetics.^1,3^

## METHODS

### Study Design, Setting, and Participants

Following IRB approval (IRB_00182712), a retrospective review was conducted of all patients who attended our institution’s RRC from January 2020 to September 2024. Men and women aged 18 years or older who received neuromodulators or soft tissue fillers during their visit to the RRC were included in the study. Patients were excluded if they had incomplete or missing clinical records.

### Resident-Run Nonsurgical Cosmetic Clinic Model

Since its implementation in 2019, our institution's RRC has allowed 1 to 2 residents per clinic to administer neuromodulator and soft tissue filler injections at a discounted rate under faculty supervision. Every Friday, residents will see 5 to 7 patients on average. Recruitment is primarily driven through patient referrals through word of mouth; however, there is also a webpage titled “Resident Cosmetic Surgery Clinic” which can be found through the University of Utah Health website describing the RRC services offered. There is no institutional advertising, social media advertising, or internal referral pathway currently in place. Our clinic utilizes a structured model to advance resident training in neuromodulator and soft tissue filler injections throughout their residency. Training begins with residents performing a minimum of 10 procedures for both neuromodulators and fillers under the guidance of attending aesthetic surgeons. This is additionally supported by biannual injection training sessions with attending aesthetic surgeons for residents of all training years. First- and second-year residents at the clinic operate under close supervision from senior residents or attendings, focusing on foundational skills and refining their techniques. Third-year residents take on a more autonomous role, independently seeing patients; however, a faculty representative is always present. Our clinic is also supported by other staff members, including a medical assistant, nurse, and clinic coordinator. Although continuity with the same resident is not guaranteed, returning patients often see the same resident when feasible.

This framework supports continuity of care, iterative learning, and a focus on patient safety.

In the resident clinic, discounted pricing is achieved primarily through reduced professional fees while maintaining equivalent product and facility costs to advanced care providers (APCs) and attendings. For neuromodulator injections, patients are charged $6.50 per unit for product, a $15 facility fee, and a flat $100 professional fee regardless of the number of units administered. For soft tissue filler injections, patients are charged a $200 professional fee, a $15 facility fee, and $150 for each additional syringe. For comparison, the APCs and attending-run clinics charge the same neuromodulator product cost ($6.50 per unit) and facility fee ($15), but higher professional fees. APC clinics charge a $5 per unit professional fee and attending clinics charge a $7 per unit professional fee. For filler injections, APC and attending-run clinics charge filler-specific product costs ranging from $400 to $510 per syringe, in addition to professional fees of $150 per syringe for APCs and $250 per syringe for attending physicians. Overall, the reduced professional fees in the RRC result in lower total out-of-pocket costs for patients compared with APC and attending-run cosmetic clinics.

During the study period, both independent and integrated residents rotated in the clinic. Historically, we accepted 3 independent residents per year, but this decreased to 1 resident in 2022, with 2024 marking the final year of accepting independent residents. Our integrated program took 2 residents annually until increasing to 3 in 2022. Given the distinct training pathways and previous experience of these groups, we analyzed their outcomes separately. Integrated residents began injectable training at postgraduate year (PGY)-1, whereas independent residents entered plastic surgery at the PGY-6 level after completing a previous residency in general surgery or another surgical specialty.

### Variables and Data Sources

Patient demographic data, treatment characteristics, and outcomes were collected from the electronic medical records system. Patient characteristics included age, gender identity, ethnicity, previous history of cosmetic injectables, and whether the patient was an established surgical patient at our institution. Treatment variables included the type of injectable used (neuromodulator, soft tissue filler, or both), injection sites, and number of units or volume injected.

Outcomes of interest included unplanned postinjectable return visits, patient retention, frequency of clinic visits, and surgical conversion rate. Unplanned postinjectable return visits were defined as clinic visits occurring within 3 weeks of the index injectable appointment and were either because of cosmetic concerns requiring revision or postinjection complications. Complications were defined as intravascular injection, infection, or ptosis. Return visits for cosmetic concerns were defined as encounters requiring revision because of perceived ineffectiveness, asymmetry, or heaviness. All cosmetic revisions were offered at no additional charge to the patient. Surgical and aesthetician consultations were assessed by identifying patients who scheduled consultations during the study period after an injectable appointment without any previous visits to these providers. Resident PGY characteristics were also documented, including the total number of residents that rotated at the clinic during the study period, number of injection encounters per PGY, and revision rates per PGY. The primary outcome measures were revision rates and complications. Secondary outcomes included patient retention rate, surgical conversion rate, injection characteristics (ie, anatomic location), and PGY characteristics. Revision rates and complications were analyzed per encounter, whereas patient retention and consultations were analyzed per patient.

### Statistical Methods

Statistical analysis was descriptive because the dataset was retrospective and longitudinal. Categorical variables were expressed as frequencies and percentages, whereas continuous variables were reported as means, ranges, and standard deviations. Individual residents contributed observations across multiple PGY levels, resulting in nonindependent observations. Additionally, the number of residents differed by PGY level. Therefore, formal inferential statistical comparisons between PGY groups were not performed. We performed all analyses using R version 3.6.1.

## RESULTS

A total of 380 patients were identified, with an average age of 41 years (Table 1). The largest age cohort was 30 to 39 years (36%), followed by 40 to 49 (23%), 20 to 29 (17%), 50 to 59 (13%), 60 to 69 (8.2%), and 70+ (2.9%). The majority of patients who visited the RRC were female (92%). The patient population was predominantly White (72%), followed by Hispanic patients (14%). Among these patients, 38% never received a neuromodulator or filler injection before visiting the RRC, 32% had received an injection from another university provider at our institution, and 40% had previously received an injection from an outside provider not at our institution. A total of 14% of patients were established surgical patients.

**Table 1.** Patient Demographics

Patient demographics	*N* = 380
Gender, *n* (%)	
Female	350/380 (92.1)
Male	30/380 (7.9)
Age, mean (SD)	41 (13)
Age distribution, *n* (%)	
<20	2/380 (0.5)
20-29	64/380 (17)
30-39	135/380 (36)
40-49	87/380 (23)
50-59	50/380 (13)
60-69	31/380 (8.2)
70-79	10/380 (2.6)
80+	1/380 (0.3)
Ethnicity, *n* (%)	
American Indian	2/380 (0.5)
AsianI	17/380 (4.5)
Bi-Racial	1/380 (0.3)
Black	5/380 (1.3)
Hispanic	52/380 (14)
Unknown/decline	28/380 (7.4)
White	275/380 (72)
First-time injection, *n* (%)	144/380 (38)
Previously injected by university provider, *n* (%)	121/380 (32)
Attending	27/121 (22)
Physician assistant-certified	34/121 (28)
Other^a^	80/121 (66)
Previously injected by outside provider, *n* (%)	150/378 (40)
Established surgical patient, *n* (%)	55/380 (14)

SD, standard deviation.

^a^Other indicates injection either by a nurse practitioner, resident, etc.

**Figure 1.**
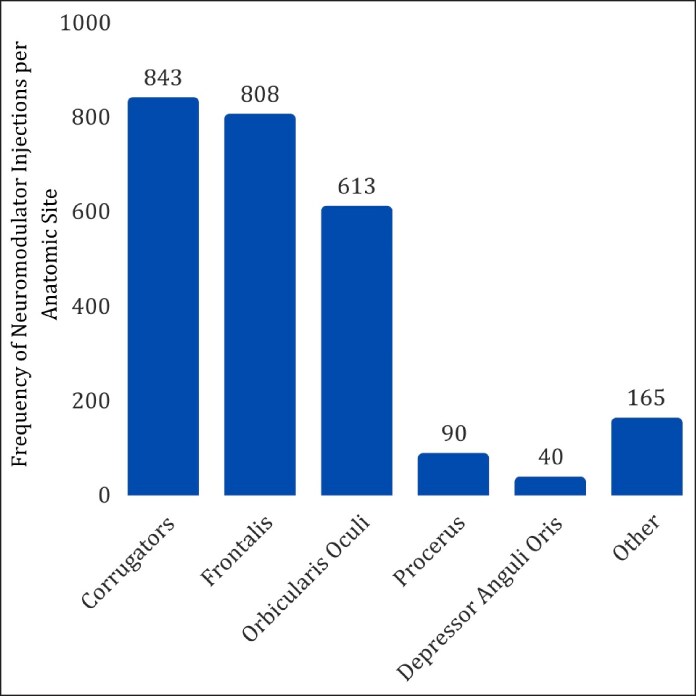
Frequency of neuromodulator injections per anatomic site during the 5-year period.

**Figure 2.**
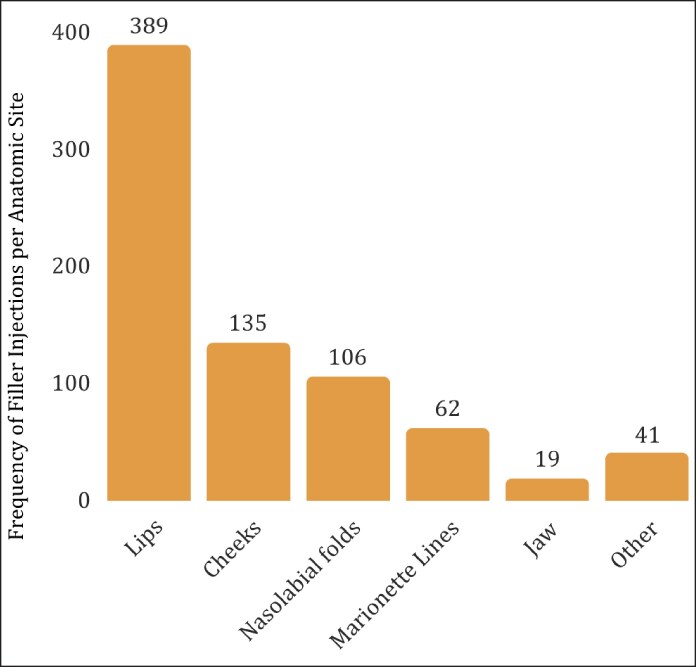
Frequency of filler injections per anatomic site during the 5-year period.

There were a total of 1168 encounters, with 55.3% involving a neuromodulator injection, 20.9% involving a filler injection, and 23.7% involving both a neuromodulator and filler injection (Table 2). A total of 923 neuromodulator injections were performed. The 5 most common neuromodulator injection sites were the corrugators (91.3%), frontalis (87.5%), orbicularis oculi (66.4%), procerus (9.8%), and depressor anguli oris (4.3%; Figure 1). A total of 522 filler injections were performed, with the 5 most common filler injection sites being the lips (74.5%), cheeks (25.9%), nasolabial folds (20.3%), marionette lines (11.9%), and jaw (3.7%; Figure 2). On average, neuromodulators were injected into 2.8 distinct anatomic sites per encounter, whereas fillers were injected into 1.5 distinct anatomic sites per encounter (Table 2). The average amount of neuromodulator and filler injected per encounter was 43 units and 1.26 cc, respectively.

**Table 2.** Summary of Encounters and Injections for Neuromodulators and Fillers

Total number of encounters	*N* = 1168
Neuromodulator only	646 (55.3%)
Filler only	245 (20.9%)
Neuromodulator and filler	277 (23.7%)
Anatomic sites injected per neuromodulator encounter	
Mean	2.8
Median	3.0
Range	1.0-5.0
Anatomic sites injected per filler encounter	
Mean	1.5
Median	1.0
Range	1.0-4.0
Neuromodulator units injected (U)	
Mean (SD)	43 (33)
Filler volume injected (cc)	
Mean (SD)	1.26 (0.85)

SD, standard deviation.

A total of 63 unplanned return-to-clinic encounters (5.4%, 63/1168) for complications or aesthetic concerns were recorded (Table 3). When analyzed by encounter type, unplanned return visit rates were 3.7% (35/923) for neuromodulator injections and 6.3% (33/522) for filler injections. These encounters involved 55 unique patients, with some returning for >1 revision. Overall, 2 true complications occurred among 1168 injectable encounters, yielding an overall complication rate of 0.17%. One patient experienced eyebrow ptosis following neuromodulator injection, which was successfully treated with an additional neuromodulator injection. One patient developed a filler-related infection, which was managed with office-based drainage and antibiotics. The remaining unplanned return visits were attributable to cosmetic concerns requiring revision, resulting in a revision rate of 5.2% (61/1168). Neuromodulator-related cosmetic revisions were most commonly because of perceived ineffectiveness (54.2%), asymmetry (31.4%), and heaviness (8.5%). Similarly, filler-related cosmetic revisions were most commonly because of perceived ineffectiveness (55.6%) and asymmetry (38.9%). One patient requested filler removal, which was successfully treated with hyaluronidase. Following these revision or complication encounters, 44 patients (84.6%) subsequently returned for routine injectable care.

**Table 3.** Unplanned Return Visits to the Resident-Run Clinics for Complications or Aesthetic Concerns^a^

Overall return-to-clinic rate by encounter	63/1168 (5.4%)
Neuromodulator return-to-clinic rate by encounter	35/923 (3.7%)
Filler return-to-clinic rate by encounter	33/522 (6.3%)
Total number of patients that returned for an aesthetic concern or complication	*n* = 55
Patient returned to the clinic after touch-up visit, *n* (%)	44 (84.6)
Total number of return-to-clinic encounters, *n* (%)	*N* = 63
Neuromodulator only	30 (47.6)
Filler only	28 (44.4)
Neuromodulator and filler	5 (7.9)
Neuromodulator reason for return, *n* (%)	*N* = 35
Asymmetry	11 (31.4)
Not effective	19 (54.2)
Heaviness	3 (8.5)
Eyebrow ptosis	1 (2.9%)
Filler reason for return, *n* (%)	*N* = 33
Asymmetry	13 (39.3)
Not effective	18 (54.5)
Infection	1 (3.0)
Filler removal	1 (3.0)

^a^A revision or complication encounter was defined as a patient returning to the clinic within 3 weeks following their previous appointment.

During the 5-year period, 37 unique residents rotated through the clinic, including both integrated and independent residents. Resident encounters and revisions by PGY level for each training model are demonstrated in Table 4. Integrated residents’ PGYs 1 to 6 were providers for 0.9%, 3.9%, 8.9%, 19.0%, 14.7%, and 8.4% of encounters, respectively. Independent residents PGYs 6 to 8 were providers for 11.7%, 15.3%, and 16.6% of encounters, respectively. The average number of clinic encounters by PGY level for integrated residents PGYs 1 to 6 was 1.4, 5.1, 9.6, 24.6, 21.5, and 14.1, respectively. For independent residents, the average number of clinic encounters for PGYs 6 to 8 was 15.2, 17.9, and 17.7, respectively. Revision rates in the integrated resident group were higher for junior residents, with PGYs 1 to 3 having revision rates of 9.0%, 8.7%, and 7.5%, respectively. Rates decreased as integrated residents advanced in training with PGYs 3 to 6 having revision rates of 7.2%, 6.2%, and 3.0%, respectively. Independent residents PGYs 6 to 8 had revision rates of 5.8%, 6.1%, and 1.5%, respectively.

**Table 4.** Resident Encounters by Postgraduate Year Level

PGY	Total encounters per PGY	Unique numbers of residents	Average encounters per resident	Median encounters per resident	Revision rate per PGY (%)
Integrated residents					
1	11	9	1.4	1.0	9.0
2	46	9	5.1	4.0	8.7
3	104	11	9.6	9.0	7.5
4	222	9	24.6	26	7.2
5	175	8	21.8	15	6.2
6	99	7	14.1	12	3.0
Independent residents					
6	137	9	15.2	14	5.8
7	179	10	17.9	15.5	6.1
8	195	9	17.7	13	1.5

PGY, postgraduate year.

Overall, 247 patients returned for at least 1 additional encounter after their initial appointment, resulting in a 65% retention rate (Table 5). On average, patients returned 2.07 times following their first visit. In terms of overall visit frequency, 35% of patients made a single visit to the clinic, 19% visited twice, 13% visited 3 times, 11% visited 4 times, and 21% visited 5 or more times. Several patients proceeded to schedule surgical consultations, with 5% (19 patients) consulting an attending physician and 4.5% (17 patients) consulting a chief resident. Additionally, 8.6% of patients scheduled a visit with a university aesthetician. Of those who pursued surgical consultations, 52.6% (10 patients) proceeded to surgery with an attending, whereas 64.7% (11 patients) proceeded to surgery with a chief resident. Overall, the conversion rate from cosmetic injection to surgery was 5.5%.

**Table 5.** Patient Retention Rates and Consultations

Patient retention rates and consultations	*N* = 380
Patient returned to the clinic at least once after the initial encounter^a^	247/380 (65%)
Average number of returns after initial encounter	2.07
Encounter frequency among patients, *n* (%)	
1	134 (35)
2	74 (19)
3	51 (13)
4	42 (11)
5+	79 (21)
Patient scheduled a surgical consult with the attending after an encounter	19/380 (5%)
Patient underwent surgery with the attending after consultation	10/19 (52.6%)
Patient scheduled a surgical consult with the chief resident after an encounter	17/380 (4.5%)
Patient underwent surgery with a chief resident after consultation	11/17 (65.7%)
Patient scheduled an aesthetician consultation after an encounter	32/380 (8.6%)

^a^The data do not include encounters for revision.

## DISCUSSION

RRCs have emerged as an integral component of plastic surgery residency education, helping to enhance residents’ cosmetic surgery training, confidence, and clinical autonomy in aesthetic care.^1-6^ Our findings expand upon the existing literature by presenting one of the largest series evaluating a nonsurgical RRC over 5 years. Our study offers new insights into how early this RRC model can optimize aesthetic training throughout residency, providing exposure to nearly 7 times the Accreditation Council for Graduate Medical Education minimum case log requirements for these procedures before graduation. In addition to demonstrating a low incidence of complications and high patient retention, our study uniquely reports on surgical conversion and long-term patient retention. These findings highlight the value of RRCs in promoting procedural competency and developing skills essential for a success in aesthetic surgery.

Over the 5-year study period, our RRC saw 380 patients for a total of 1168 encounters. On average, patients were 41 years old, with 92.1% of the cohort being female and 7.9% male. These findings are consistent with national trends reported by the ASPS and patient demographics seen at other RRCs for injectable use.^1,13,15^ Interestingly, patients 30 to 39 years old comprised the largest portion of our patient population (36%), followed by those aged 40 to 49 (23%) and 20 to 29 (17%). Cosmetic injectable use has been increasing among patients in their 20's and 30's, representing a paradigm shift in the nonsurgical cosmetic landscape among millennials.^15,17^ This trend seen in our results may also be influenced by the demographics of our RRC with younger residents who are uniquely positioned to create a more relatable and comfortable environment, attracting a younger patient population. Additionally, offering injectables at a discounted rate may further increase accessibility to cosmetic care. Consistent with previous reports on the ethnic distribution of cosmetic procedures, Caucasian and Hispanic individuals made up the 2 largest cohorts of patients undergoing cosmetic injectables, with smaller contributions from Asian and African American patients.^18^

Within our study cohort, 55.3% of encounters involved neuromodulator injections alone, whereas 20.9% and 23.7% involved filler injections and combination treatments, respectively. The most common anatomic locations for neuromodulator and filler injections described in our study align with previous studies describing RRCs’ injectable use.^1,12,19^ Notably, Bagwell et al found that their RRC had comparable patient volume and injectable exposure compared with an attending cosmetic clinic.^12^ This suggests that nonsurgical RRCs offer a training environment that can closely replicate independent practice settings.

Of the 1168 encounters, 5.4% resulted in a return to the clinic for either a complication or aesthetic concern. Interestingly, these revisions involved 55 patients (13.6% of the total patient cohort), indicating that certain patients were more likely to request touch-ups or further adjustments. This may be because of higher aesthetic expectations, differential responses to treatment, or underlying psychological factors that influence patients’ perception of their outcome. The most common reason for return was asymmetry or perceived lack of effectiveness. Asymmetry was most commonly reported in the eyebrows, forehead, or lips which are typical sites for injection-related asymmetry.^19,20^ One patient experienced eyebrow ptosis, corresponding to an incidence of 0.10%. This rate is substantially lower than the reported incidence of eyebrow ptosis, occurring in 1% to 5% of neuromodulator cases on average.^21-23^ Our total infection rate (0.19%) is in line with what has been reported in the literature (0.04%-0.2%).^24^ Walker et al reported an injectable revision rate of 4% at their RRC, whereas Qureshi et al found that no patients required a revision following injectables.^1,13^ However, both studies are limited by a small sample size (23 and 45 patients, respectively). Nonetheless, these findings demonstrate that nonsurgical RRCs generally demonstrate low revision rates and complications, which our study outcomes further bolster with an increased sample size. Overall, these findings suggest that training residents in injectable procedures is both safe and effective, with a low incidence of complications and outcomes that are comparable to national data.

During the study period, both integrated and independent residents rotated in the clinic. Our clinic's graduated model of autonomy is reflected in the results, with residents PGY-3 and above performing the majority of the procedures. Quong et al and Weissler et al demonstrated that longitudinal exposure to aesthetic procedures is associated with improved perceived competence and confidence in residents performing various aesthetic surgical procedures.^11,25^ Our study further supports this finding, demonstrating a notable trend within the integrated resident model toward decreased revision rates as residents advanced in their training. Notably, Quong et al identified limited exposure and lower self-reported competence in nonsurgical facial procedures.^11^ In our RRC model, the early introduction of injectable training at the PGY-1 level allows residents to refine their skills, leading to proficiency over time and improved competency in injectables by their chief year. Of note, our PGY-1s did have a lower average encounter of 1.4 with the highest revision rate of 9.0%; this may be attributed to the smaller sample size skewing the revision rate or decreased surgical experience before entering the clinic. Given the revision rate is increased compared with the other PGY cohorts, this beckons further investigation into potential benefit from additional training specific to injectables for PGY-1 residents before entry into the clinic. Independent residents (PGY 6-8) had revision rates comparable to those of integrated residents at the PGY 5-6 level. This is likely because of their previous surgical experience before entering the program. However, although their independent residents enter with a higher baseline proficiency, integrated residents achieve similar competency by their final years. These findings highlight the effectiveness of early procedural exposure and a graduated autonomy model to optimize resident competency and outcomes with injectables.

Retention rates at our RRC were high, with 65% of patients returning to the clinic at least once following their initial encounter, with 45% of our patient cohort visiting the clinic 3 or more times. Importantly, of those who underwent a revision procedure, 84.6% returned to the clinic for routine injectable care. Our clinic's retention rate mirrors those described in independent practice settings.^26,27^ Fino et al found that among 429 patients who received nonsurgical rejuvenation procedures in a private practice, 55% of patients still returned to the clinic 18 months following their treatments.^26^ Similarly, White et al reported a patient return rate ranging from 55% to 67% 6 months after their injectable procedures at their independent practice.^27^ Although we did not directly measure patient-reported experience or outcomes, our high retention rates suggest that patients who visit the RRC have a positive experience with the services they have received. A study by Qureshi et al and Iorio et al demonstrated statistically significant improvements in patient-reported outcome measures before and after receiving neuromodulator and filler injections at their RRC using the FACE-Q scale.^1,28^ These studies demonstrate the beneficial role of RRCs in delivering safe, high-quality care for nonsurgical cosmetic procedures. Additionally, we evaluated the clinic's conversion rate from nonsurgical to surgical procedures and found that 21 patients progressed to surgical intervention (5.5% conversion rate). Despite the limited data on conversion rates, many studies have demonstrated that noninvasive aesthetic treatments can serve as an entry point to invasive cosmetic procedure.^29-31^ Our results may further support this concept and highlight that RRCs can serve as a valuable pathway for residents to develop skills that are important for practice, including patient acquisition, recruitment, and retention. However, there is a need for studies directly comparing conversion rate from RRCs, academic clinics, and private practice settings.

### Limitations

Although our study has several strengths, it is not without its limitations, and our results should be interpreted with those in mind. Our study reflects the experience of a single institution with data collected retrospectively, which may be influenced by confounding factors. Retrospective data collection can also result in incomplete information, potentially affecting the accuracy results. Not all complications may have been fully captured, because patients may have sought treatment for adverse events from another institution or practice rather than at the RRC, and given the retrospective nature of the study, we were unable to assess for immediate complications such as edema, bruising, nodularity, Tyndall effect, or vascular compromise without necrosis, which may have influenced our low complication rate. Additionally, because of the retrospective nature of the study, standardized measures of satisfaction such as FACE-Q surveys were not implemented at the time of treatment or follow-up requiring us to extrapolate satisfaction from retention rates comparable to the literature. Furthermore, the lack of comparative data with other RRCs or attending clinics makes it difficult to assess how our clinic's outcomes and practice volume compare with other practice settings. Finally, a single-institution experience limits the ability to generalize the results to other plastic surgery programs that may have a different program structure and patient demographics.

## CONCLUSIONS

Nonsurgical RRCs are useful training tools, offering longitudinal exposure to nonsurgical facial rejuvenation procedures while maintaining high-quality care without compromising patient safety. Our study demonstrates that nonsurgical RRCs effectively integrate residents across all levels of training, achieve high patient retention, and maintain complication rates comparable to those reported in independent practice settings, while offering greater economic accessibility for patients. Further studies evaluating long-term patient satisfaction, resident satisfaction, PGY-1 training before RCC participation, and cost-effectiveness may help further evaluate the efficacy of this clinic model.
